# The soluble VCAM-1 level is a potential biomarker predicting severe acute graft versus host disease after allogeneic hematopoietic cell transplantation

**DOI:** 10.1186/s12885-022-10096-3

**Published:** 2022-09-20

**Authors:** Sook-Kyoung Heo, Eui-Kyu Noh, Yoo Jin Lee, Yerang Shin, Youjin Kim, Hyeon-Su Im, Hyeyeong Kim, Su Jin Koh, Young Joo Min, Jae-Cheol Jo, Yunsuk Choi

**Affiliations:** 1 Biomedical Research Center, Ulsan University Hospital, University of Ulsan College of Medicine, Ulsan, Republic of Korea; 2 Department of Hematology and Oncology, Ulsan University Hospital, University of Ulsan College of Medicine, 877 Bangeojinsunhwan-doro, Dong-gu, Ulsan, 44033 Republic of Korea; 3 Department of Hematology, Asan Medical Center, University of Ulsan College of Medicine, 88, Olympic-ro 43-gil, Songpa-gu, Seoul, Seoul 05505 South Korea

**Keywords:** Soluble VCAM-1, Acute graft versus host disease, Biomarker, Allogeneic stem cell transplantation

## Abstract

**Background:**

Severe graft versus host disease (GVHD) is the main reason for non-relapse mortality following allogeneic hematopoietic cell transplantation (HCT). We investigated the serum protein profiles of patients who had undergone HCT to identify predictive biomarkers of severe acute GVHD (aGVHD).

**Methods:**

Serum samples were collected for 30 patients from day − 7 to day + 14 of HCT. The serum levels of plasma beta2-microglobulin (β2-MG), soluble vascular cell adhesion molecule-1 (sVCAM-1), platelet factor 4, and TNFSF-14 were measured by ELISA as potential biomarkers following 310 cytokine profiling array.

**Results:**

The median age of the study patients was 53.5 years (range, 19–69). All grade and grade 2–4 aGVHD developed in 21 (70.0%) and 17 (56.7%) patients, respectively. Compared with their baseline levels on day − 7, β2-MG and sVCAM-1 were significantly increased on day + 14 of the HCT procedure (*P* = 0.028 and *P* < 0.001, respectively). Patients with a grade 2–4 severe aGVHD showed a significantly higher sVCAM-1 level at baseline (day-7) and at day + 14, compared with the other group with a grade 1 aGVHD or no aGVHD (*P* = 0.028 and *P* = 0.035, respectively).

**Conclusion:**

Higher sVCAM- levels at baseline and on day + 14 in HCT patients could be a significant predictive biomarker of severe aGVHD.

**Supplementary Information:**

The online version contains supplementary material available at 10.1186/s12885-022-10096-3.

## Introduction

Allogeneic hematopoietic cell transplantation (HCT) is the most effective therapeutic modality for hematologic malignancies and bone marrow failure syndrome [1]. Graft versus host disease (GVHD) is the main cause of early and late transplantation-related mortality (TRM) in these patients [2, 3]. Acute GVHD (aGVHD) is a condition in which the donor T cells attack host tissues including the skin, liver, and gastrointestinal tract [4, 5]. Although GVHD prophylaxis approaches including calcineurin inhibitors, tacrolimus or cyclosporine, and methotrexate, have been routinely administered in HCT, the incidence of GVHD still ranges from 30 to 50% following this therapy [4, 6]. As grade 2 to 4 aGVHD is life-threatening, a high dose (2 mg/kg/day) of methylprednisolone therapy is indicated as the initial treatment [5]. GVHD and its treatment may cause damage to the barrier function and also result in immunosuppression, leading to a higher risk of infectious disease [7, 8]. About 50% of patients with severe aGVHD do not respond to first-line treatments, and the 1-year survival rate of patients with steroid-refractory aGVHD is less than 20% [4]. Hence, the early prediction of high-risk patients who may develop severe GVHD is an important issue for HCT. For the past 20 years, various groups have been investigating potential biomarkers, including IL-2Rα (CD25), IL-7, TNFR1, regenerating islet-derived protein-3 alpha (REG3α), ceruloplasmin, ST2, sBAFF and miR-155, and miR-586 to enable an earlier and more accurate diagnosis, and improved risk stratification, of patients with aGVHD [4, 9–11]. However, the data on potential biomarkers for GVHD have been heterogeneous to date, according to the study groups, and too complex to apply to any clinical setting. To date also, no single cytokine or panel has been clearly defined as a useful biomarker of aGVHD. The identification of biomarkers predicting severe GVHD could lead to earlier detection of severe GVHD before its clinical manifestation, which could in turn reduce the TRM rate and thus improve post-transplantation outcomes. Therefore, we aimed to investigate serum protein profiles of patients who had undergone HCT to find predictive biomarkers for severe aGVHD.

## Methods

### Patient samples

We included 30 patients with a hematologic malignancy or bone marrow failure syndrome who had undergone HCT at the Ulsan University Hospital in Korea between 2017 and 2019. Cell-free serum samples had been collected weekly on days − 7, 0, + 7, and + 14 (pre- and post-HCST) from these subjects. All included patients gave informed consent for blood collection and analysis of their clinical data, and this study was performed in accordance with the Declaration of Helsinki and approved by the Ulsan University Hospital Institutional Review Board (UUH 2017–06–006-003).

### Cytokine profiling antibody arrays

When selecting patient samples for analysis via a cytokine profiling antibody array, we initially selected two representative cases (one with and one without aGVHD) with acute myeloid leukemia (AML) with similar characteristics i.e., female, aged above 60, and with a complete remission status after induction and consolidation chemotherapy. One of these patients developed grade 3 aGVHD post-HCT, and the other had no GVHD. To screen potential biomarkers of aGVHD, a cytokine profiling array containing 310 proteins was performed using serum samples of two patients collected on day + 14 post-HCT.

The cytokine profiling antibody array experiment was performed according to the manufacturer’s instructions [12–14]. Sample preparation was conducted by the manufacturer’s protocol. Proteins were extracted using a commercially available buffer (Full Moon Biosystems, Sunnyvale, CA, USA) containing a 1% protease inhibitor cocktail (Sigma, St. Louis, MO, USA), 1% phosphatase inhibitor cocktail (Sigma, St. Louis, MO) and lysis beads (Full Moon Biosystems, Sunnyvale, CA, USA). Following this extraction, the protein solution was purified using a gel matrix column that was included in the antibody array assay kit (Full Moon Biosystems, Sunnyvale, CA, USA). The column was mixed by vortexing for 5 seconds and hydration-treated for 60 minutes at room temperature. After hydration, the column was centrifuged at 750 x g for 2 minutes and placed into a collection tube. The 100 μl protein sample was then loaded onto the column, which was then centrifuged at 750 x g for 2 minutes. The concentration of the purified sample was measured using a BCA protein assay kit (Pierce, Rockford, IL, USA) and a NanoPhotometerTM (Implen, UK). The purity of the sample was confirmed on the UV spectrum.

According to the manufacturer’s instructions for the cytokine profiling antibody array experiment [12–14], 50 μg of the protein samples were dissolved in 75 μl of labeling buffer and mixed with 3 μl of 10 μg/μl biotin/DMF solution. Then, the samples which were incubated for 90 min at room temperature were mixed with 35 μl of stop reagent and incubated at room temperature for 30 min. 30 ml of blocking solution in a petri dish was added to each antibody microarray slide (Full Moon Biosystems, Sunnyvale, CA, USA), which was incubated on a shaker at 60 rpm for 30 min at room temperature, and washed with distilled water. This step was replicated three times. The blocked array slide was rinsed with Milli-Q grade water. The labeled sample was mixed in 6 ml of coupling solution. In a coupling dish, the array slide with a coupling mixture was incubated on a shaker at 60 rpm for 2 hours at room temperature. Then, the slide was washed six times with 30 ml of washing solution into a petri dish on a shaker at 60 rpm for 5 minutes and rinsed with Milli-Q grade water. A 30 μl aliquot of 0.5 mg/ml Cy3-streptavidin (GE Healthcare, Chalfont St. Giles, UK) was next mixed in 30 ml of detection buffer. The coupled array slide in a petri dish on a shaker was treated with the detection mixture at 60 rpm for 20 minutes at room temperature. Then, the slide was washed six times with 30 ml of washing solution at 60 rpm for 5 minutes and rinsed with Milli-Q grade water [12–14].

### Cytokine profiling antibody array data acquisition and analysis

To obtain data from the cytokine antibody arrays, each slide was completely dried and scanned within 24–48 hours at a 10 μm resolution, optimal laser power, and photomultiplier tube (PMT) using a GenePix 4100A scanner (Axon Instruments Inc., Union City, CA, USA). After capturing the scanned image, the signals in each grid on the slide were evaluated and quantified with GenePix 7.0 Software (Molecular Devices Corporation, Sunnyvale, CA, USA). The obtained protein information was annotated using UniProt database (https://www.uniprot.org). The fold changes in the serum cytokine levels were compared between the samples with or without aGVHD. Briefly, the serum protein profile was analyzed as the ratio between serum protein levels of the grade 3 aGVHD case (65-year-old female with AML) and the patient without aGVHD (61-year-old female with AML) at the day + 14 timepoint post-HCT.

### Cytokine level measurements using ELISA

Cell-free plasma from the peripheral blood samples of the 30 study patients that had undergone HCT was collected and frozen at − 80 °C. We then measured the beta 2-microglobulin (β2-MG), soluble vascular cell adhesion molecule-1 (sVCAM-1), platelet factor 4 (PF4), and tumor necrosis factor superfamily member 14 (TNFSF-14) levels in samples collected from the study patients on days − 7 and + 14 before and after HCT. β2-MG, sVCAM-1, PF4, and TNFSF-14 levels were also analyzed in samples collected from 10 patients on days 0 and day + 7 using an ELISA kit, in accordance with the manufacturer’s instructions (R&D Systems, Minneapolis, MN).

### Allogeneic hematopoietic cell transplantation procedures

The hematopoietic stem cell donors were determined via an HLA typing match through the DNA-sequencing of the HLA-A, −B, −C, and -DR loci. The conditioning regimens for HCT were selected at the discretion of the treating clinicians, who took account of the disease status and the patient’s general condition in each case. Myeloablative conditioning (MAC) included Bu4Cy (intravenous busulfan at 3.2 mg/kg/day for 4 days on days − 7 to − 4 and cyclophosphamide at 60 mg/kg/day for 2 days on days − 3 and − 2) and Bu4Flu (intravenous busulfan at 3.2 mg/kg/day for 4 days on days − 7 to − 4 and fludarabine at 30 mg/m^2^/day for 6 days on days − 7 to − 2). Twenty-eight patients received rabbit anti-thymocyte globulin (ATG; Thymoglobulin, Genzyme Transplant) at a total dose range of 4.5 to 9 mg/kg on days − 4 to − 2. Reduced-intensity conditioning (RIC) regimens included Bu3FluATG (intravenous busulfan at 3.2 mg/kg/day from days − 7 to − 5, fludarabine at 30 mg/m^2^/day from days − 7 to − 2, and ATG at 1.5–3 mg/kg/day from days − 4 to − 2), Bu2FluATG (intravenous busulfan at 3.2 mg/kg/day on days − 7 and − 6, fludarabine at 30 mg/m^2^/day from days − 7 to − 2, and ATG at 1.5–3 mg/kg/day from days − 4 to − 2), CyFluATG (cyclophosphamide at 60 mg/kg/day on days − 3 and − 2, fludarabine at 30 mg/m^2^/day from days − 6 to − 2, and ATG 3 at mg/kg/day from days − 4 to − 2) and FluATG (fludarabine at 30 mg/m^2^/day from days − 7 to − 2 and ATG at 3 mg/kg/day from days − 4 to − 2). For graft-versus-host disease (GVHD) prophylaxis, 28 patients received cyclosporine, and two patients received tacrolimus. Methotrexate was given to all 30 patients for 3 to 4 days in addition to cyclosporine or tacrolimus. A bone marrow graft was given in 1 patient (3.3%) and peripheral blood hematopoietic stem cells in 29 patients (96.7%) on day 0 or from days 0 to 1. Granulocyte colony-stimulating factor (450 μg/day) treatment commenced from day 5 and continued until the day of an absolute neutrophil count over 3000/μL. Thirteen patients received veno-occlusive disease (VOD) prophylaxis using prostaglandin.

### Diagnosis of acute graft versus host disease

The diagnosis of aGVHD was generally based on careful clinical evaluations in all patients and additional biopsies of the involved sites in some cases. AGVHD was graded by the modified Glucksberg criteria [15]. Data for baseline clinicopathological features, treatments, and acute and chronic GVHD were collected from the medical records of each patient.

### Statistical analysis

The cytokine levels between samples were compared using the nonparametric Mann-Whitney U-test or Kruskal-Wallis test. Statistical analyses were performed using SPSS version 21.0 software (IBM Corp., Armonk, NY). For all analyses, the *P* values were 2-tailed, and *P* < 0.05 (*P* < 0.01 for Spearman’s rank correlation analysis) was considered statistically significant. We calculated the GVHD incidence, the cumulative incidence of relapse (CIR), and non-relapse mortality (NRM) rates using cumulative incidence estimates, adjusting for competing risks.

## Results

### Demographic and clinical characteristics of the study patients

**Fig. 1:**
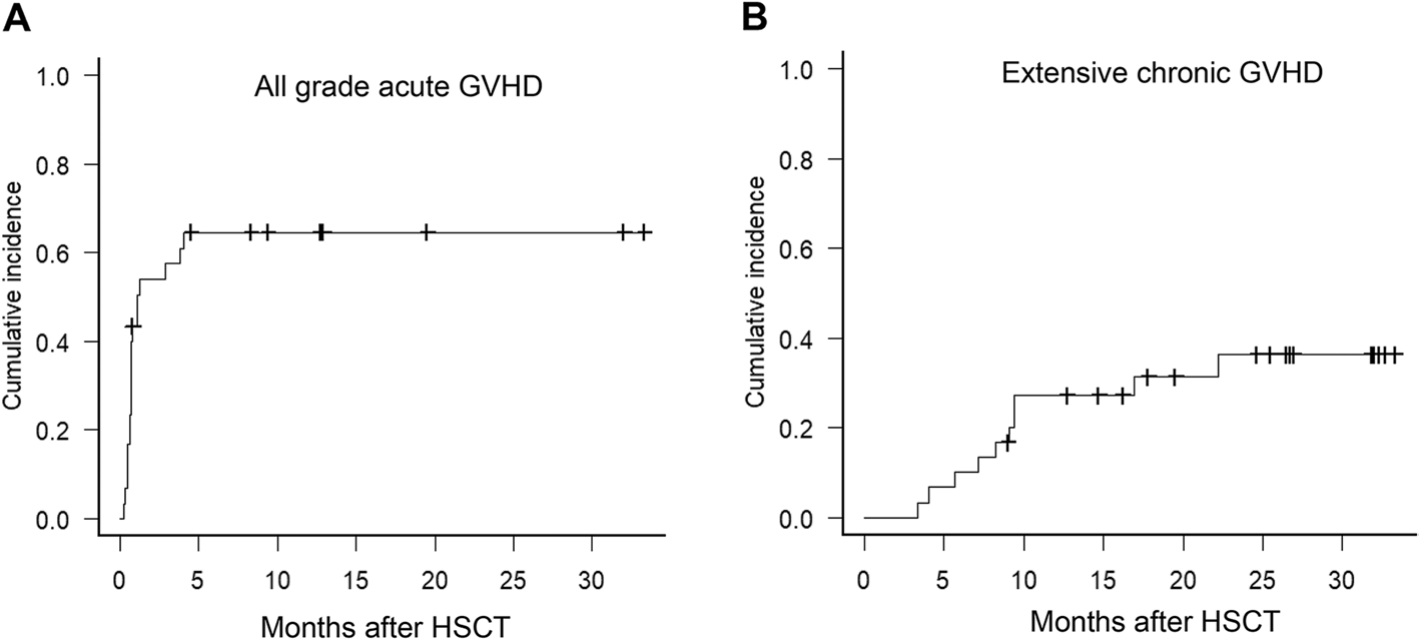
Cumulative incidence of (**A**) all grades of acute graft versus host disease (GVHD) and (**B**) extensive chronic GVHD

Study patient characteristics are summarized in Table 1. The median age was 53.5 years (range, 19–69) and 46.7% of these subjects were male. The disease distribution among this cohort included AML (*n =* 9), acute lymphoblastic leukemia (ALL) (*n =* 6), myelodysplastic syndrome (MDS) (*n =* 7), aplastic anemia (AA) (*n =* 4), and myelofibrosis (MF) (*n =* 4). Seventeen patients (56.7%) were in CR status, 12 (40.0%) of patients with MDS, AA, or MF, and one patient (3.3%) with AML had the persistent disease at the time of HCT. Myeloablative conditioning regimens were used in 5 patients (16.7%) and reduced-intensity conditioning was administered in 25 patients (83.3%). Except in two patients, ATG was administered at a total dose range of 4-9 mg/kg. Patients received HCT from matched sibling donors (*n =* 11), HLA-matched unrelated donors (*n =* 9), mismatched unrelated donors (*n =* 5), or haploidentical family donors (*n =* 5). The median CD34+ cell dose was 6.3 ×  10^6^/kg (range, 2.0–18.8 × 10^6^/kg). The male donors were 63.3%, and the median donor age was 32.5 years (range, 23–61 years). The cumulative incidences of all grade aGVHD and extensive chronic GVHD are provided in Figs. 1A and B. All grade aGVHD developed in 21 patients (70.0%), and grade 2–4 aGVHD arose in 17 cases (56.7%). Chronic GVHD (cGVHD) and extensive cGVHD were found in 56.7 and 33.3% of the patients, respectively.

**Table 1:** Characteristics of the study patients who underwent allogeneic hematopoietic cell transplantation (*n =* 30)

Variables	No. of patients (%)
Sex, male/female	16 (46.7%)/ 14 (53.3%)
Age, year, median (range)	53.5 (19–69)
Disease	
AML	9 (30.0%)
ALL	6 (20.0%)
MDS	7 (23.3%)
AA	4 (13.3%)
Lymphoma	2 (6.7%)
PMF	2 (6.7%)
Disease status at HSCT	
Complete remission	17 (56.7%)
Persistent disease (MDS, PMF, AA)	12 (40.0%)
Primary refractory status	1 (3.3%)
Conditioning intensity	
Myeloablative conditioning	5 (16.7%)
Bu4Cy + ATG	4 (13.3%)
Bu4Flu + ATG	1 (3.3%)
Reduced intensity	25 (83.3%)
Bu3Flu + ATG	8 (26.7%)
Bu2Flu + ATG	12 (40.0%)
CyFlu+ATG or Flu+ATG	5 (16.7%)
Total dose of ATG	
No use	2 (6.7%)
4.5 mg/kg	8 (26.7%)
6 mg/kg	5 (16.7%)
9 mg/kg	15 (50.0%)
Donor type	
MSD	11 (36.7%)
URD (HLA matched / mismatched)	14 (9 / 5) (46.7%)
Familial haplo-identical	5 (16.7%)
HLA disparities	
0	20 (66.7%)
1	4 (13.3%)
2	1 (3.3%)
4	5 (16.7%)
Graft source	
Bone marrow	1 (3.3%)
Peripheral blood stem cell	29 (96.7%)
Cell dose, median (range)	
TNC, × 10^8^/kg	8.8 (2.0–20.5)
MNC, × 10^8^/kg	5.3 (0.6–14.5)
CD34+, × 10^6^/kg	6.3 (2.0–18.8)
Donor sex, male/female	19 (63.3%)/ 11 (36.7%)
Donor age, years, median (range)	32.5 (23–61)
GVHD prophylaxis	
Cyclosporin + methotrexate	28 (93.3%)
FK506+ methotrexate	2 (6.5%)
Acute GVHD, all grade	21 (70.0%)
Grade 1	4 (13.3%)
Grade 2	11 (36.7%)
Grade 3–4	6 (20.0%)
Grade 2–4 acute GVHD, involved organ	17 (56.7%)
Skin	12
Liver	7
Gastrointestinal tract	7
Chronic GVHD, all grade	17 (56.7%)
Limited	7 (23.3%)
Extensive	10 (33.3%)

*AML* Acute myeloid leukemia, *ALL* Acute lymphoblastic leukemia, *MDS* Myelodysplastic syndrome, *AA* Aplastic anemia, *PMF* Primary myelofibrosis, *HSCT* Allogeneic hematopoietic stem cell transplantation, *Bu4Cy* Four days of busulfan and two days of cyclophosphamide, *Bu4Flu* Four days of busulfan at 3.2 mg/kg/day and six days of fludarabine at 30 mg/kg/day, *Bu2Cy* Two days of busulfan at 3.2 mg/kg/day and two days of cyclophosphamide, *Bu2Flu* Two days of busulfan at 3.2 mg/kg/day and six days of fludarabine at 30 mg/kg/day, *ATG* Anti-thymocyte globulin, *HLA* Human leukocyte antigen, *MSD* Matched sibling donors, *URD* Unrelated donors, *Haplo* Familial haploidentical donors, *TNC* Total nucleated cells, *MNC* Mono-nucleated cells, *GVHD* Graft-versus-host disease

### Cytokine profiling antibody array

**Fig. 2:**
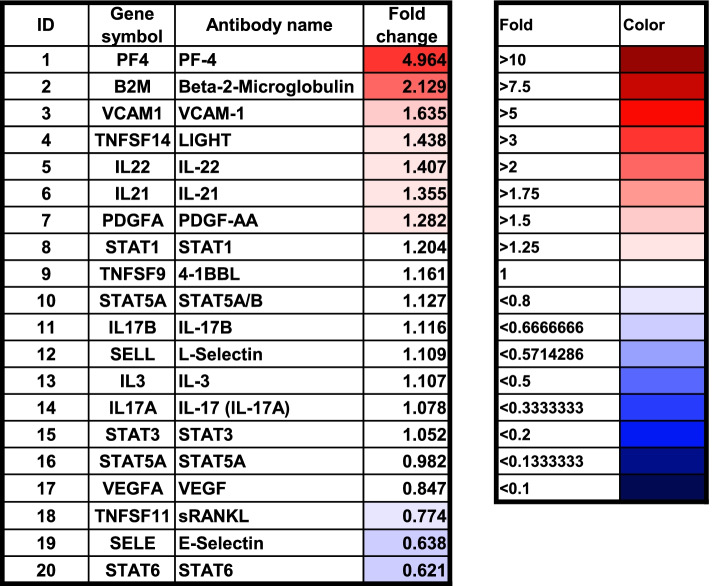
Differences in the serum protein levels may be associated with the frequency of post-HCT acute graft versus host disease (GVHD). The serum protein profile was analyzed between a patient with grade 3 acute GVHD (a 65-year-old woman with AML) and a patient without acute GVHD (a 61-year-old woman with AML) on day + 14 after HCT

Cytokine profiling array data using post-HCT (day + 14) samples of two representative patients with grade 3 severe aGVHD and no GVHD are shown in Fig. 2. Noticeable fold changes were evident in PF4, beta 2-microglobulin (β2-MG), sVCAM-1, TNFSF-14, IL-22, IL-21, and PDGFA in the severe aGVHD patient. Among the 310 proteins analyzed in total on the cytokine profiling array (Supplement data 1.), PF4 (fold change, 4.964), β2-MG (fold change, 2.129), sVCAM-1 (fold change, 1.635), and TNFSF-14 (fold change, 1.438), all elevated in the post-HCT day+ 14 sample of the severe aGVHD patient, were selected as potential biomarkers for severe aGVHD.

### Changes in the potential cytokine aGVHD biomarkers, β2-MG, sVCAM-1, PF-4, and TNFSF-14 during HCT

The changes in the levels of the four serum cytokines, β2-MG, sVCAM-1, PF-4, and TNFSF-4 which were selected as potential biomarkers of aGVHD and measured at baseline (day − 7), day 0, day 7, and day + 14 post-HCT, are listed in Table 2. Compared with the baseline, the serum β2-MG and sVCAM-1 level were significantly increased on day + 14 after HCT (*P* = 0.028 and *P* < 0.001, respectively). On the other hand, the PF4 levels were significantly decreased on day + 14 compared with day − 7. The TNFSF-14 levels were unchanged between day-7 (baseline) and day+ 14.

**Table 2:** Cytokine levels at day −7, day 0, day + 7 and day + 14 of HSCT

Cytokine level, ng/ml, mean + SE	Time after HSCT				*P*-value^a^ (between day − 7 and day 0)	*P*-value^a^ (between day − 7 and day + 7)	*P*-value^a^ (between day − 7 and day + 14)	*P*-value^a^ (between day + 7 and day + 14)
	day − 7 (*n =* 30)	day 0 (*n =* 10)	day + 7 (*n =* 10)	day + 14 (*n =* 30)				
β2- MG	4429.2 ± 514.4	1223.3 ± 126.6	5960.1 ± 689.7	5651.3 ± 492.5	0.959	0.386	**0.028**	0.878
sVCAM-1	1039.4 ± 78.7	1223.3 ± 126.6	1194.0 ± 132.2	1614.7 ± 88.8	0.445	0.799	**< 0.001**	**0.007**
PF4	406.5 ± 27.9	286.2 ± 61.7	236.2 ± 44.6	313.9 ± 25.7	0.508	0.284	**0.006**	0.508
TNFSF-14	33.7 ± 3.0	30.4 ± 6.2	21.4 ± 1.3	29.0 ± 2.4	0.858	**0.017**	0.130	**0.047**

*HSCT* Allogeneic hematopoietic stem cell transplantation, *β2- MG* Beta2-microglobulin, *sVCAM-1* Soluble VCAM-1, *SE* Standard error of the mean

^a^Wilcoxon signed-rank test

### Differences in the β2-MG, sVCAM-1, PF-4, and TNFSF-14 levels between patients with severe (grade 2–4) acute GVHD and those with grade 1 acute or no GVHD

**Fig. 3:**
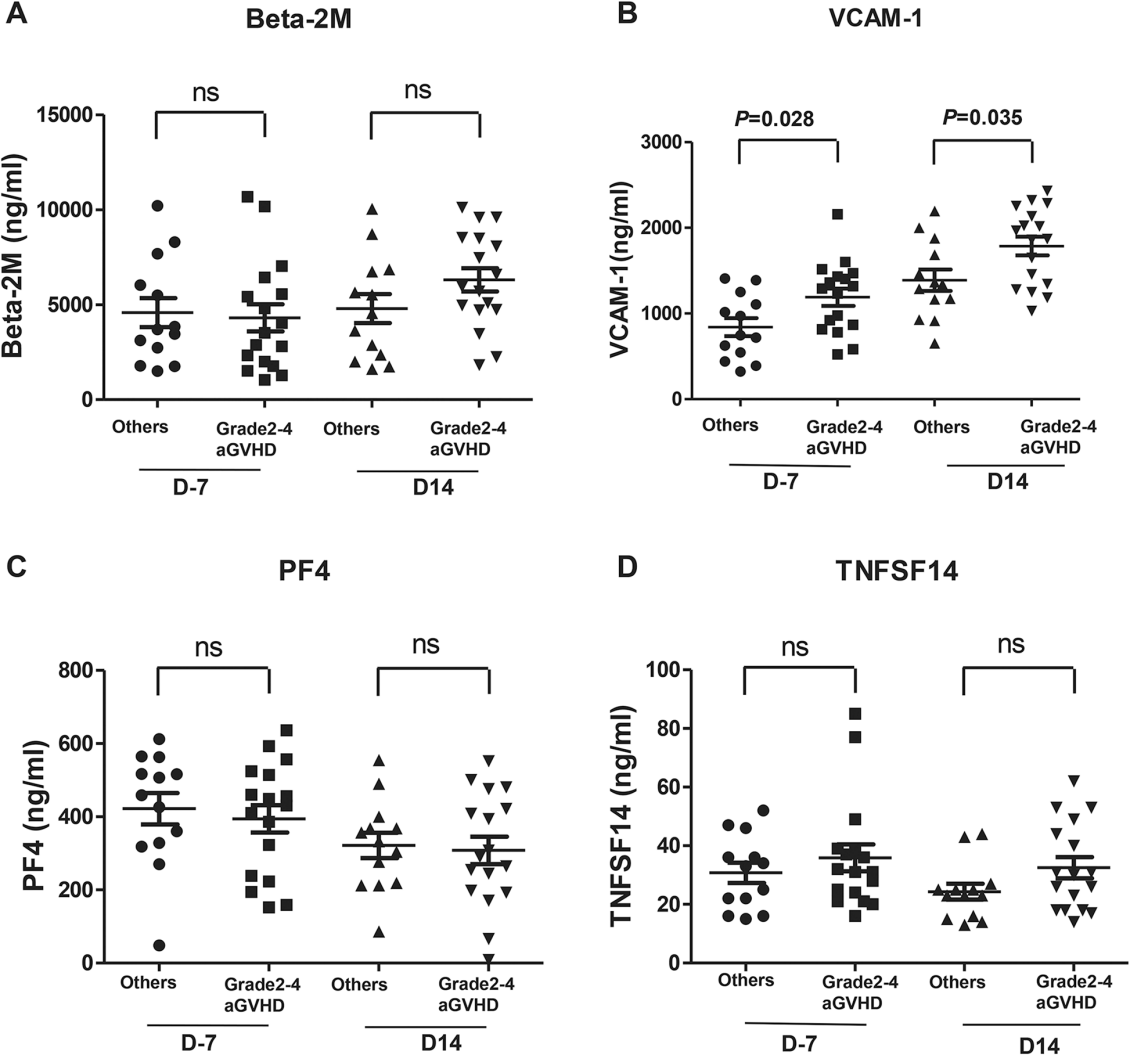
Serum levels of cytokines according to the development of grade 2–4 acute graft versus host disease. **A** Beta2-microglobulin. **B** sVCAM-1. **C** PF4. **D** TNFSF-14

**Fig. 4:**
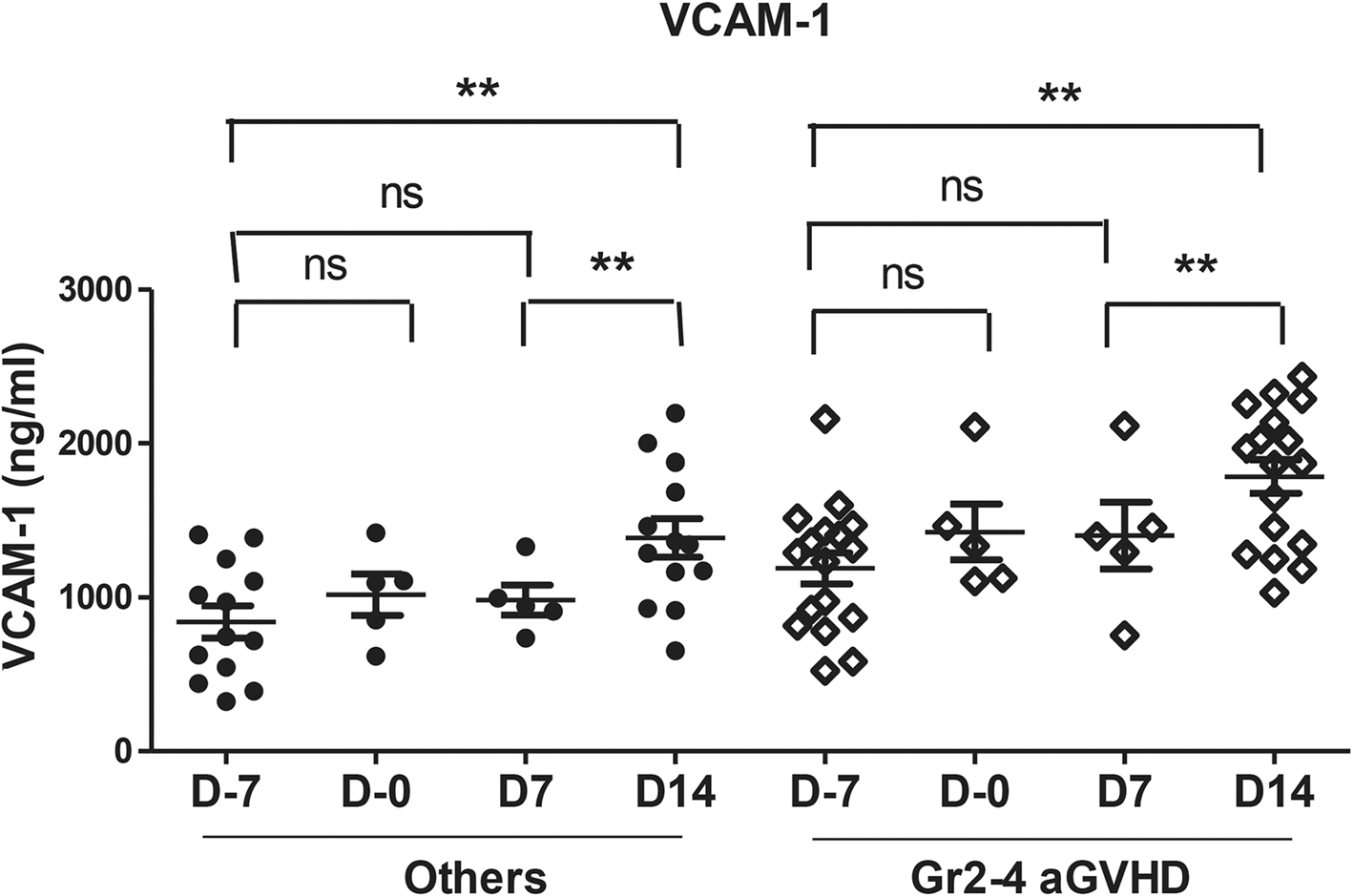
Changes in the serum sVCAM-1 level according to the time point (days − 7, 0, + 7, and + 14) in relation to allogeneic hematopoietic cell transplantation

The β2-MG, sVCAM-1, PF-4, and TNFSF-14 levels measured on day-7 (baseline) and day+ 14 after HCT were compared in terms of the onset of severe (grade 2–4) aGVHD (Table 3). The soluble VCAM levels on day-7 (mean, 1191.2 ng/ml vs. 841.0 ng/ml; *P* = 0.028) and day + 14 (mean, 1787 ng/ml vs. 1389.1 ng/ml; *P* = 0.035) were significantly higher in the severe (grade 2–4) aGVHD cases than in those with no aGVHD or only grade 1 aGVHD (Fig. 3). While the sVCAM-1 level showed an increasing pattern from day − 7 to day + 14 post-HCT, this was more prominent in the severe (grade 2–4) aGVHD group (Fig. 4). However, there were no significant differences in β2-MG, PF4, or TNFSF-14levels between severe (grade 2–4) aGVHD and the other groups on day-7 and day+ 14 samples (Fig. 3).

**Table 3:** Differences in the cytokine levels at day −7 and day + 14 of HSCT between the grade 2–4 acute GVHD group and the other group (*n =* 30)

	day − 7^a^			day + 14^a^		
Cytokine, mean ± SE	Acute GVHD, grade 2–4 (*n =* 17)	Others^b^ (*n =* 13)	*P*-value*	Acute GVHD, grade 2–4 (*n =* 17)	Others^a^ (*n =* 13)	*P*-value*
β2- MG, ng/ml	4311.8 ± 712.9	4582.8 ± 765.5	0.711	6306.8 ± 617.4	4794.1 ± 761.5	0.145
sVCAM-1, ng/ml	1191.2 ± 101.3	841.0 ± 104.3	**0.028**	1787 ± 109.3	1389.1 ± 125.3	**0.035**
PF4, ng/ml	394.4 ± 37.4	422.2 ± 43.1	0.563	308.0 ± 37.7	321.6 ± 34.6	0.805
TNFSF-14, ng/ml	35.9 ± 4.6	30.8 ± 3.5	0.621	24.3 ± 2.7	32.5 ± 3.6	0.086

*HSCT* Allogeneic hematopoietic stem cell transplantation, *GVHD* Graft versus host disease; *β2- MG* Beta2-microglobulin, *sVCAM-1* Soluble VCAM-1, *SE* Standard error of the mean

^a^The timepoint of the serum cytokine measurement in relation to the HSCT

^b^Included patients with no acute GVHD or grade 1 acute GVHD

*Mann-Whitney U test

According to the involved organ in the aGVHD cases such as the skin (*n =* 12), gastrointestinal tract (*n =* 7), and liver (*n =* 7), no significant differences were found in the sVCAM-1 level between day − 7 (*P* = 0.328, *P* = 0.133, *P* = 0.270, respectively) and day + 14 (*P* = 1.0, P = 1.0, P = 0.270, respectively).

### ROC curve analysis of sVCAM-1 level on D-7 and D14 after HCT for the development of grade 2–4 acute GVHD

**Fig. 5:**
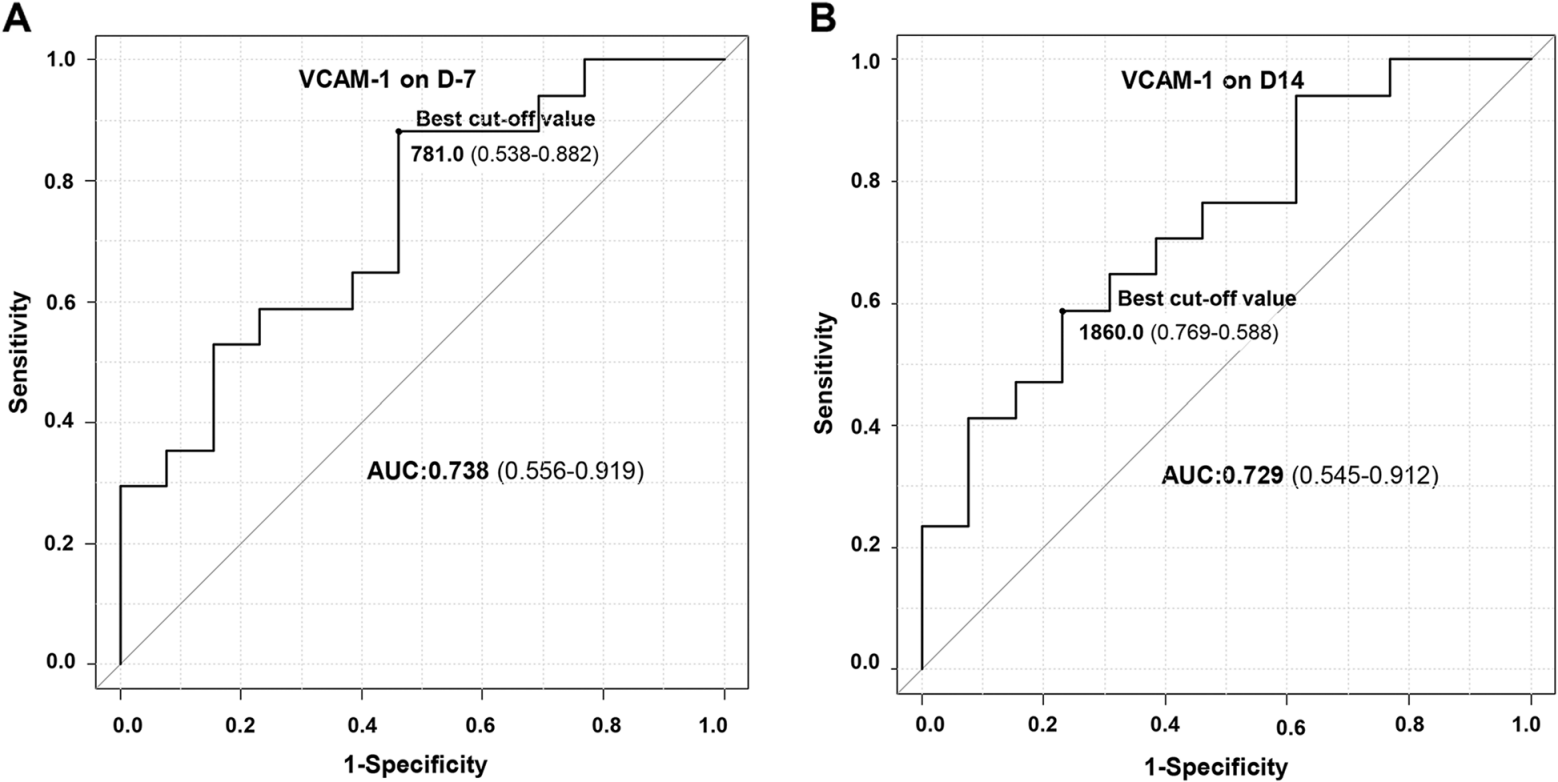
ROC curve analysis of sVCAM-1 level (ng/ml) on D-7 (**A**) and on D14 (**B**) for the development of grade 2–4 acute graft versus host disease. AUC, area under the curve

In ROC curve analysis for the development of grade 2–4 aGVHD, the area under the curve (AUC) of sVCAM-1 level on D-7 (baseline) and D14 was 0.738 (97% CI, 0.556–0.919) and 0.729 (0.545–0.912), respectively (Fig. 5). The best cut off value of sVCAM-1 was 781 ng/ml (95% CI, 0.538–0.882) on D-7 and 1860.0 (95% CI, 0.769–0.588) on D14 after HCT. For grade 2–4 aGVHD, the positive predictive value and negative predictive value of sVCAM-1 were 71.4 and 77.8% on D-7 and 76.9 and 58.8% on D14, respectively. The sensitivity and specificity of sVCAM-1 were 71.4 and 77.8% on D-7 and 76.9 and 58.8% on D14, respectively.

### Differences in the β2-MG, sVCAM-1, PF-4, and TNFSF-14 levels between patients with extensive chronic GVHD and those with limited chronic GVHD or no GVHD

There were no significant differences in the serum β2-MG, sVCAM-1, PF-4, and TNFSF-14 levels on day − 7 and day + 14 between the extensive chronic GVHD and no or limited chronic GVHD cases (Table 4).

**Table 4:** Differences in the cytokine levels at day -7 and day + 14 for HSCT between the chronic extensive GVHD group and the other group

	day -7^a^			day +14^a^		
Cytokine, mean ± SE	Extensive chronic GVHD (*n =* 10)	Others^b^ (*n =* 20)	*P*-value*	Extensive chronic GVHD (*n =* 10)	Others^b^ (*n =* 20)	*P*-value*
β2- MG, ng/ml	4488.3 ± 842.7	4399.7 ± 661.4	0.746	5559.5 ± 525.7	5697.3 ± 699.6	0.983
sVCAM-1, ng/ml	1171.7 ± 154.7	973.3 ± 88.5	0.328	1770.8 ± 144.1	1536.7 ± 110.4	0.214
PF4, ng/ml	389.2 ± 50.7	415.1 ± 34.1	0.681	360.3 ± 45.5	290.7 ± 30.6	0.198
TNFSF-14, ng/ml	36.9 ± 5.2	32.1 ± 3.7	0.286	32.3 ± 4.8	27.3 ± 2.8	0.307

*HSCT* Allogeneic hematopoietic stem cell transplantation, *GVHD* Graft versus host disease, *SE* Standard error of the mean

^a^The timepoint of the serum cytokine measurement in relation to the HSCT

^b^Others included patients with limited chronic graft versus host disease (GVHD) and those with no chronic GVHD

*Mann-Whitney U test

## Discussion

The development of the most severe GVHD following HCT has remained unpredictable, and this can lead to fatal clinical situations. The prediction and prevention of severe GVHD are crucial in reducing the NRM rate and increasing the cure chance following HCT. The median onset of aGVHD is 1 month after HCT, [16] but Moon et al. have reported that the early onset aGVHD can occur from as early as 3 days to 27 days (median, 18 days) post- HCT, which is associated with a poorer survival outcome [17]. Hence, the identification of effective biomarkers that can detect aGVHD in the earlier periods of HCT is clinically useful and meaningful. A recent study by Solan et al. reported that the elafin plasma levels at day + 15 were higher in patients with severe skin aGVHD and suggested that this could be a predictive biomarker for skin aGVHD in a haploidentical HCT [18]. However, since serum elafin was measured on day 15 and day 30 after HCT, and the onset time of aGVHD and the timing of serum elafin measurement can overlap, the clinical use of elafin as a predictive biomarker before the development of aGVHD might be difficult.

The pathophysiology of acute GVHD is associated with alloreactive T cell activation and systemic inflammation [19]. Therefore, among the 310 proteins, we prioritized and analyzed the proteins related to the inflammatory response. We tried to select novel markers that had not been well evaluated in previous studies regarding GVHD biomarkers. For validation, we excluded some markers whose clinical significance to GVHD was already assessed in the previous studies or whose mechanisms seem not to be relevant to predicting the occurrence of GVHD. As a result, PF4, β2-MG, VCAM-1, and TNFSF-14 were prioritized for validation in the present study. PF4 is a small cytokine belonging to the CXC chemokine family that promotes blood coagulation and plays a role in wound repair and inflammation [20]. β2-MG, which has been used as a prognostic factor for blood cancers such as multiple myeloma and lymphoma, was a interesting result. In addition, there have been some reports that the VCAM-1 and TNFSF-14 proteins are related to the inflammatory response [21–23].

In our present study, a higher sVCAM-1 level at baseline before HCT and on day 14 after HCT was found to be a potential predictive marker of the risk of grade 2–4 severe aGVHD. In our study patients who developed grade 2–4 aGVHD, the baseline serum sVCAM-1 level was significantly higher than in those with grade 1 or no aGVHD. Moreover, the sVCAM-1 level significantly continued to increase from the baseline to the day + 14 after HCT both in the severe aGVHD group and the other group. ROC curve analysis of sVCAM-1 level also revealed that sVCAM-1 on D-7 and D14 is a predictive marker (AUC, 0.738 and 0.729, respectively) for predicting the development of grade 2–4 acute GVHD. The best cut off value of sVCAM-1 was 781 ng/ml (95% CI, 0.538–0.882) on D-7 and 1860.0 (95% CI, 0.769–0.588) on D14 after HCT. For the prediction of acute grade 2–4 GVHD, the positive predictive value and the sensitivity of sVCAM-1 were higher on D14 than on D-7. However, the negative predictive value and specificity of sVCAM-1 were higher on D-7 than on D14. Of particular note in this regard, as sVCAM-1 upregulation can be detected in the baseline serum sample before administering the conditioning regimen, it has clinical utility as a predictive biomarker. Hence, a risk prediction for severe aGVHD through the measurement of the serum sVCAM-1 level in the earlier phase of HCT can potentially be used to develop risk-adapted GVHD prophylaxis strategies.

sVCAM-1 (CD106) is a 90-kDa glycoprotein and is one of the cell adhesion molecules included among the members of the immunoglobulin superfamily [24]. sVCAM-1, along with intercellular cell adhesion molecules 1,2 and 3 (ICAM-1,2, and − 3) and platelet endothelial cell adhesion molecule-1 (PECAM-1; CD31), forms part of a group of functionally related cell adhesion molecules that function as critical mediators in endothelial cell and leukocyte/connective tissue interactions and control directed leukocyte migration across microvascular endothelial barriers [25–27]. These cell adhesion molecules, including sVCAM-1, can be detected in plasma by the proteolytic cleavage of the membrane-bound counterparts from activated leukocytes and endothelial cells and/or by the differential splicing of their transcripts [27–29]. Pro-inflammatory cytokines, such as TNFα, one of the critical cytokines in the pathogenesis of GVHD, enhance VCAM-1 expression [24, 30, 31]. In a previous study using an in vivo mouse model with GVHD of the central nervous system (CNS), Mathew et al. demonstrated that VCAM-1 expression of the endothelial cell was downregulated by selective TNF gene deletion in the microglia of these mice [32]. However, there have been only a few data on the clinical implications of sVCAM-1 in GVHD after HCT. In a study by Eyrich et al., upregulation of VCAM-1, ICAM-1, B7–1, and B7–2 was demonstrated in an allogeneic mouse model with GVHD on day 22, whereas other adhesion molecules, such as ICAM-2, platelet-endothelial cell adhesion molecule 1, E-selectin and mucosal addressin cell adhesion molecule 1 were not changed [33]. In our data, sVCAM-1 level was not found to be organ-specific for the involved sites of aGVHD such as skin, gastrointestinal tract, and liver, but seemed to be associated with the severity of aGVHD. The elevated sVCAM-1 level at day + 14 was also not related to the development of extensive chronic GVHD, based on our current data. However, a higher level of sVCAM-1 at day 60 after HCT has been reported to be associated with chronic GVHD [34].

Prior to HCT, most patients with hematologic malignancies received intensive chemotherapies for remission induction and consolidation, which may cause tissue inflammation and injury. This may lead to various sVCAM-1 level increases in accordance with the degree of tissue inflammation and the patient’s vulnerability to chemotherapy. VCAM-1 contains an extracellular domain with six or seven immunoglobulin-like domains, some of which bind ligands including α4β1 integrin and α4β7 integrin [35, 36]. sVCAM-1 could thus be a potential therapeutic target in aGVHD. As a supporting example of this, the binding of α4β1 integrin to VCAM-1 can be inhibited by natalizumab, which targets the α4 integrin, a key mediator of lymphocyte trafficking, and produces non-selective anti-inflammatory effects [37]. Recently, natalizumab combined with corticosteroid has been investigated as an initial treatment for grade 2–3 gastrointestinal aGVHD in a phase II trial and has shown efficacy with a 52% overall response rate at 56 days [38].

The upregulation of VCAM-1 expression in the endothelia of grafted organs has also been reported to be associated with graft rejection in which lymphocytes and monocytes play a central role. There have been several investigations trying anti-VCAM-1 monoclonal antibody in murine models in vivo as a therapeutic approach to improve allograft survival [39, 40]. Although monoclonal antibiotics blocking VCAM-1 have not been tested to date as a treatment for aGVHD, anti VCAM-1 monoclonal antibody may be a potential therapeutic agent for aGVHD, based on the present data.

The present study had several limitations of note, such as the small number of included patients. In cytokine profiling array to screen the biomarkers, a comparison of one aGVHD patient with another no GVHD patient could increase the risk of selecting the wrong marker (such as β2-MG, PF-4, and TNFSF-14) and missing the best biomarker for GVHD.

Our data on the utility of sVCAM-1 as a predictive biomarker also need to be further validated. However, our present findings have clinical value as we have conducted a relatively widescreen via a cytokine profiling array of 310 proteins to compare severe aGVHD and mild or no aGVHD patients. The serum sVCAM-1 level was subsequently found to be highly expressed in patients with severe aGVHD at preconditioning baseline and day+ 14 post-HCT. There have been few studies about sVCAM-1 as an aGVHD biomarker. In the future, we need to conduct a large-scale multicenter study to validate sVCAM-1 and a combination of sVCAM-1 and multiple biomarkers, such as ST2, REG3α, TNFR1, and IL-2Rα for the prediction of severe aGVHD.

## Conclusion

A higher sVCAM-1 level at preconditioning baseline and at day+ 14 after HCT is a potentially useful biomarker to predict the development of severe aGVHD in the early period of HCT. Larger-scale clinical trials are needed for validation.

## Supplementary Information

**Additional file 1.**

## Data Availability

The datasets generated and/or analyzed during the current study are available in the gene expression omnibus (GEO) in NCBI repository (GEO accession number: GSE205842, https://www.ncbi.nlm.nih.gov/geo/query/acc.cgi?acc=GSE205842).
